# Supplementary material for: Association between liberal oxygen therapy and mortality in patients with paraquat poisoning: A multi-center retrospective cohort study
Source: PLoS One. 2021 Jan 15;16(1):e0245363. doi: 10.1371/journal.pone.0245363 (PMC7810293; doi:10.1371/journal.pone.0245363)
Supplement: Attachment — Submitted filename: Response to reviewers.docx [file pone.0245363.s003.docx]

Response to the Editor and the Reviewer

Dear Academic Editor: Muhammad Adrish

Thank you for giving us the opportunity to submit the revision of “The association between liberal oxygen therapy and mortality in patients with paraquat poisoning: a multi-center retrospective cohort study” for your consideration for publishing in the PLOS ONE.

Thanks to the reviewers for their careful review. The reviewers’ comments have been carefully read and responded as following.

We hope the finding and conclusion described in our manuscript may benefit patients who got paraquat intoxication worldwide, and we also hope the precious readers of PLOS ONE will find this study interesting. We are hopeful that this manuscript will meet the standards of your prestigious journal and be favorably reviewed.

We look forward to hearing from you again soon.

Sincerely yours,

Corresponding author:

Dr. Po-Chun Chuang, MD

Department of Emergency Medicine

Kaohsiung Chang Gung Memorial Hospital

No. 123, Dapi Rd., Niaosong Dist. Kaohsiung 833, Taiwan, R.O.C.

E-mail: zhungboqun@gmail.com.

*Dear reviewer #1:*

1. *I note you still present the fact that no association between liberal oxygen and outcome in paraquat poisoning has been presented before. I do not think this is true and both reviewers provided multiple references to show this.*

**Response:** Thank you for your kind reminder. The sentence has been removed.

2. *Line 183 - signing DNR consents, - this should be something like "**patients with DNR status" Suggest changing throughout*

**Response:** Thank you for your kind reminder. There are thee “signing DNR consents” in our article and we have changed them to “patients with DNR status”.

3. *Line 228 - pulmonary function (not s) by causing cytotoxic reactions.*

**Response:** Thank you for your friendly reminder. The sentence has been rewritten to make it clearer.

4. *Line 231 - Conclusion: Oxygen therapy should be administered with caution and should be reserved for those with hypoxia (SpO2 < 90 %)." Isn't that what happened??? Oxygen requirement is a signal for poor outcome.*

**Response:** Thank you for your kind reminder. It is true that oxygen requirement is a signal for poor outcome. In addition, this study revealed the association between the liberal oxygen therapy and mortality in patients with paraquat poisoning. However, this study could only reveal the correlation, not causation. The causation between liberal oxygen therapy and mortality need to be proved by further prospective studies and randomized control trials. However, the ethical and legal issues restrict the conduction of prospective studies since oxygen therapy are harmful for paraquat poisoning patients. To make it clear, the conclusion was rewritten as “Unless the evidence of hypoxia (SpO2 < 90%) is clear, oxygen therapy should be avoided because it is associated with increased mortality.”

*Reviewer #2: (No Response)*
